# Hair Follicle Bulge Stem Cells Appear Dispensable for the Acute Phase of Wound Re‐epithelialization

**DOI:** 10.1002/stem.2289

**Published:** 2016-02-02

**Authors:** Clare L. Garcin, David M. Ansell, Denis J. Headon, Ralf Paus, Matthew J. Hardman

**Affiliations:** ^1^ The Healing Foundation Centre, Faculty of Life SciencesUniversity of ManchesterManchesterUnited Kingdom; ^2^ Institute of Inflammation and RepairUniversity of ManchesterManchesterUnited Kingdom; ^3^ The Roslin Institute and Royal (Dick) School of Veterinary StudiesUniversity of EdinburghEdinburghUnited Kingdom; ^4^ Department of DermatologyUniversity of MünsterMünsterGermany

**Keywords:** Skin, Hair follicle, Bulge, Wound healing

## Abstract

The cutaneous healing response has evolved to occur rapidly, in order to minimize infection and to re‐establish epithelial homeostasis. Rapid healing is achieved through complex coordination of multiple cell types, which importantly includes specific cell populations within the hair follicle (HF). Under physiological conditions, the epithelial compartments of HF and interfollicular epidermis remain discrete, with K15^+ve^ bulge stem cells contributing progeny for HF reconstruction during the hair cycle and as a basis for hair shaft production during anagen. Only upon wounding do HF cells migrate from the follicle to contribute to the neo‐epidermis. However, the identity of the first‐responding cells, and in particular whether this process involves a direct contribution of K15^+ve^ bulge cells to the early stage of epidermal wound repair remains unclear. Here we demonstrate that epidermal injury in murine skin does not induce bulge activation during early epidermal wound repair. Specifically, bulge cells of uninjured HFs neither proliferate nor appear to migrate out of the bulge niche upon epidermal wounding. In support of these observations, Diphtheria toxin‐mediated partial ablation of K15^+ve^ bulge cells fails to delay wound healing. Our data suggest that bulge cells only respond to epidermal wounding during later stages of repair. We discuss that this response may have evolved as a protective safeguarding mechanism against bulge stem cell exhaust and tumorigenesis. Stem Cells *2016;34:1377–1385*

**Significance Statement**

In response to skin injury, peri‐wound hair follicles become activated to make a significant contribution to the healing process. Until recently, the bulge region, which houses the best‐characterized population of stem cells within the hair follicle, has been considered the major contributor to wound repair. Our research contradicts this widely accepted paradigm and reveals bulge inactivity during the first stages of wound healing. This may represent an evolutionarily programmed mechanism to protect against injury‐induced cancer formation.

## Introduction

The hair follicle (HF) is a dynamically remodeled miniorgan comprising multiple adult stem cell (SC) populations that underlie its tremendous growth and regeneration potential 1. In homeostasis, the HF and interfollicular epidermis (IFE) are maintained exclusively by two distinct populations: the cycling HF epithelium by bulge epithelial HF SCs, which divide infrequently, contributing to the outer root sheath (ORS), inner root sheath, hair matrix, and secondary hair germ; the IFE by basal epidermal SCs, which divide more frequently to replenish terminally differentiating keratinocytes 2, 3, 4, 5, 6. While the keratin 15^+ve^ bulge is considered the primary HF SC niche, housing “the most quiescent and long‐lived” population of cells 5, more recently identified SC populations also exist within the HF, such as the Lgr6 and MTS24 positive isthmus region 7, 8, and the Lrig1^+ve^ junctional zone 9.

Under specific conditions of perturbed epidermal homeostasis, HF‐derived keratinocytes actively contribute to the IFE. For example, following injury HF‐derived keratinocytes proliferate and migrate into the neo‐epidermis (IFE) 2, 3, 7, 9, 10, 11. Interestingly, cells deriving from different HF regions may make varying contributions to epidermal wound healing. For example, Page et al. 9 have extensively characterized the contribution of numerous HF cell populations, including the bulge, and have shown that Lrig1^+ve^ cells persist for up to a year in the healed IFE, whereas Ito et al. 3 showed depletion of HF‐derived cells of the K15^+ve^ lineage within 20 days postwounding. Studies have also demonstrated that HF contribution may be important for healing rate immediately after wounding. Specifically, Langton et al. 12 showed a delay in the early stages of tail skin healing in the absence of HFs, while we have more recently demonstrated accelerated skin repair during the anagen phase of the hair cycle 13.

However, the temporal kinetics of the HF‐derived cell contribution to epidermal healing remains unclear. It is particularly important to understand the identity and location of the cell populations that are first activated and responsible for the early contribution of HFs to wound repair. Conversely, whether specific HF progenitor cell niches might be dispensable for epidermal repair remains unknown. The latter issue is particularly important in light of recent work demonstrating (a) plasticity both within and between HF SC niches 14 and (b) the role of K19^+ve^ cells in HF regeneration and healing 15.

In this study we clarify the role of K15^+ve^ bulge cells in the early stages of epidermal repair. Here we present the first detailed analysis of HF keratinocyte proliferation over an extensive early wound healing time course. While many studies have analyzed the involvement of HFs in wound re‐epithelialization during the later stages (7 days onwards) to our knowledge until now, the kinetics of the early HF response to wounding has not been studied. We show that while upper HF ORS keratinocytes rapidly proliferate following epidermal injury, with differential induction kinetics between anagen and telogen HFs, K15^+ve^ bulge cells are not activated to proliferate by epidermal injury and appear to be largely dispensable for timely wound re‐epithelialization. We suggest that these unexpected findings reveal an evolutionarily conserved mechanism to protect against tumorigenesis.

## Materials and Methods

### Animal Experimentation

All animal procedures were approved by the University of Manchester ethical review panel and performed under UK home office licence (number 40/3020 and 70/8136). Mice were housed in groups prior to experimentation with access to food and water ad libitum.

### Generation and Treatment of Transgenic Mice

The previously described K15Cre^PR^ transgenic strain 16 was crossed with the previously described strain containing the Diphtheria toxin A fragment (DTA) with an upstream floxed stop codon 17 (C57BL/6 genetic background). Prior to transgene activation, mice were healthy, bred normally, and had no obvious phenotype. Transgene induction was performed at 7 weeks of age to ensure that all HFs were within the telogen phase of hair cycle 18. Topical induction with 2 µg RU486 per 1 mg Neutrogena hand cream (50 µl cream applied per 1.5 cm mouse skin) (Sigma, Poole, U.K.) was performed daily for 5 days prior to wounding (Supporting Information Fig. S1A) 16 and continued after wounding to maintain depletion of bulge cells. Mice were culled at 3 days post injury.

### Wounding Procedure

Male C57BL/6 mice at either 32 days old (anagen) or 49 days old (telogen) were anaesthetized with isofluorane by inhalation. A single 6 mm full thickness excision was made on the left hand side of the dorsal flank, with the right hand side left unwounded as an internal contralateral control (Supporting Information Fig. S1B). The control was used to identify any systemic wound induced changes to hair cycle (none were observed). Buprenorphine was administered for analgesia, and wounds were left to heal by secondary intention, with mice being housed individually postoperatively.

K15cre^PR^;DTA mice and sex‐matched littermate controls were anaesthetized, and 2 × 6 mm full thickness excisions were made on the dorsal flank. The biopsy tissue obtained at the time of wounding was collected and fixed for histology, to confirm efficacy of bulge ablation treatment at the time of injury (i.e., the amount of cell death within the bulge was analyzed). The two wounds per animal were assessed as independent replicates 18.

### Tissue Harvesting

Mice were culled by rising concentration of CO_2_ at 2, 4, 6, 12, 18, 24, or 72 hours postwounding. All mice received 40 mg/kg of 5‐bromo‐2‐deoxyuridine (BrdU) via i.p. injection 2 hours prior to cull to assess proliferation within a defined period (Supporting Information Fig. S1C), with the exception of the continual proliferation study which received BrdU at the time of wounding and subsequently every 12 hours until cull at 72 hours (or every 12 hours from 108 hours until cull at 7 days) (Supporting Information Fig. S1D). All wounds were bisected along the caudal to rostral plane to present HFs in a longitudinal orientation (Supporting Information Fig. S1B). Bisected wounds were fixed and processed as previously described 13. Contralateral control skin was also taken for the C57BL/6 experiment in order to confirm that the hair cycle was identical in unwounded regions of skin. For K15cre^PR^ DTA studies, controls tissue was taken from the biopsy (time zero), plus also treated and untreated regions at the time of cull.

### Immunohistochemistry

Approximately 5 μm tissue sections were used for immunohistochemistry. Protocols for BrdU, Keratin 6, and Cd34 have been previously described 13. Keratin 15 immunohistochemistry was performed via the ABC method using the mouse on mouse kit (Vector; Peterborough, U.K., http://www.vectorlabs.com). Antigen retrieval was via the citrate (pH 6) method with primary antibody incubation performed at 4°C for 16 hours (1:50; AbCam, Cambridge, U.K., http://www.abcam.com). Sections were counterstained with Gills haematoxylin (Vector). TUNEL assay was performed using the in situ cell death detection kit (Roche, Basel, Switzerland, http://www.roche‐applied‐science.com) as per manufacturer's instructions, antigen retrieval with Proteinase K (Sigma) at 20 μg/ml for 20 minutes at 37°C. Keratin 15 CD34 and α6 integrin were quantified as number of outer bulge cells present in control and K15cre^PR^;DTA mice.

### Quantitative Immunohistomorphometry of Proliferating HF Cells

**Figure 1:**
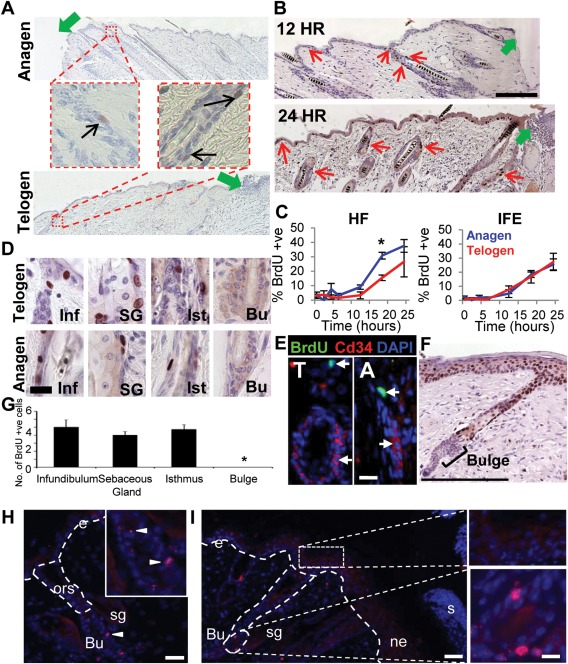
Hair follicle proliferation is rapidly induced in response to injury, but the bulge neither proliferates nor migrates during the first 3 days of healing. Male C57/Bl6 mice were wounded in either anagen or telogen to profile the early injury induced proliferative response. **(A)**: Proliferation within the HF is observed as early as 12 hours postwounding, as shown by BrdU incorporation assay (black arrows). Green arrows denote wound edge. **(B)**: HF proliferation propagates outward from the wound edge (BrdU positive cells labelled by red arrows), green arrows denote wound edge. **(C)**: Anagen HFs show a more rapid proliferative response to wounding compared to telogen HFs, significant at 18 hours postwounding, while no difference is observed in the inter follicular epidermis (IFE). **(D)**: BrdU^+ve^ cells were observed in all HF regions, except for the bulge, in both anagen and telogen, confirmed by an absence of BrdU/CD34 dual immunofluorescence colocalization **(E). (F)**: Continuous BrdU administration throughout the 3 days of healing also confirms an absence of bulge proliferation. **(G)**: Quantification of proliferating cells within different HF regions (24 hours postinjury). **(H)**: BrdU immunohistochemistry showing bulge label retention in unwounded skin. Dashed line indicates basement membrane; e, interfollicular epidermis; ors, outer root sheath; sg, sebaceous gland; Bu, bulge. Inset shows an enlarged image of the bulge region, arrow heads show label retaining cells. **(I)**: BrdU immunofluorescence of 3 day wound tissue. S, scab; ne, neoepidermis; e, interfollicular epidermis; Bu, bulge. Enlarged images show label retaining cells in the bulge, and an absence of label retaining cells in the epidermis. Mean ± SEM, *n* = 3–5. *, *p* < .05; scale bar = 50 µm (B), 25 µm (D), 25 µm (E), 200 µm (F), 30 µm (H, I). Abbreviations: Bu, bulge; BrdU, 5‐bromo‐2‐deoxyuridine; DAPI, 4′,6‐diamidino‐2‐phenylindole; HF, hair follicle; Inf, interfollicular epidermis; SG, sebaceous gland.

Wound edge tissue sections were analyzed for BrdU incorporation within the basal layer of the IFE (excluding neo‐epidermis) and cells within the ORS of HFs. HFs that had been partially amputated during wounding were excluded from analysis. In order to directly compare the same cellular material from anagen VI HFs we only counted ORS within the upper HF (proximal to the bulge). Supporting Information Figure S1E indicates zones of analysis, with BrdU counts presented as a % of total measureable basal cells in that region. Quantification of BrdU positive cells per HF region is presented as a raw number (Fig. 1G).

### Pulse‐Chase Label Retention Assay

Label retention assay was performed as described by Taylor et al. 11. C57BL/6 WT mice were subcutaneously injected with BrdU labeling reagent (50 µg/g b.wt.) twice daily between P3 and P5 (for 3 days). Mice were wounded at P23 and sacrificed 3 days later at P26 (Supporting Information Fig. S1F).

### Colocalization Immunofluorescence Studies

Dual immunofluorescence of BrdU and Cd34 was conducted on paraffin‐embedded tissue, with antigen retrieval via the citrate method. Sections were blocked with rabbit serum before incubating for 16 hours with primary antibody at 4°C (BrdU 1:50, Abcam; Cd34 1:100, Santa Cruz, Santa Cruz, CA, http://www.scbt.com). Sections were washed with Phosphate buffered saline (PBS) supplemented with 0.1% Tween‐20, then incubated in secondary antibody (Donkey anti Rat‐alexa 488 1:100, Invitrogen, Carlsbad, CA, http://www.invitrogen.com; Donkey anti goat‐Rhodamine 1:100, Millipore, Billerica, MA, http://www.millipore.com). Sections were washed, counterstained with DAPI, and mounted.

### Statistical Analysis

Statistical analysis across multiple wounding time points was performed using a one‐way ANOVA with post hoc testing. Analysis of bulge ablation experiment was via pairwise Student's *t* test.

## Results

### Injury Rapidly Induces Peri‐Wound HF Proliferation that Propagates Radially from the Wound Edge

To address the temporo‐spatial kinetics of the early HF response to epidermal injury, we profiled the behavior of noninjured HFs adjacent to full thickness skin wounds (hereby termed peri‐wound HFs), during the first 24 hours postinjury. Our methodology, using a single 6 mm circular excision, permitted the first detailed proliferation analysis of longitudinally sectioned wound edge HFs, with corresponding unwounded contra‐lateral flank skin (See Supporting Information Fig. S1 for experimental design). These analyses revealed that injury induces upper ORS keratinocyte proliferation in peri‐wound HFs from 6 hours postwounding in both anagen and telogen HFs (Fig. 1A). Moreover, induced proliferation emanated from the wound edge (Fig. 1B), suggesting that inductive signals are wound‐derived rather than intrinsic to the HF.

### Anagen Hs Proliferate More Rapidly in Response to Injury

Given that the hair cycle phase strongly influences wound healing outcome 13, we hypothesized that the effects of wounding on HF proliferation would be hair cycle‐dependent. Comparing hair‐cycle‐synchronized mouse skin fields 19, 20 revealed a more rapid onset of proliferation in the ORS of peri‐wound anagen HFs (growth phase) than telogen HFs (resting phase; Fig. 1C). Of note, no hair cycle‐dependent difference in proliferation was observed in the wound edge IFE (Fig. 1C), suggesting that hair cycle does not directly influence the IFE response to injury.

### Peri‐Wound HFs do not Display Bulge Proliferation

Curiously, extensive analysis of HF proliferation during the first 72 hours postinjury revealed an absence of bulge localized proliferation, despite BrdU^+ve^ cells in virtually all other HF compartments (Fig. 1D, 1G). Importantly, cellular colocalization of BrdU and the bulge marker CD34 was never observed (Fig. 1E). To confirm that we had not simply missed a narrow window of bulge proliferation, an additional cohort of mice received BrdU injections every 12 hours from wounding until collection 72 hours later. In these mice, virtually all IFE and distal ORS cells were BrdU^+ve^, while all bulge cells remained BrdU^−ve^ (Fig. 1F, 1G). This finding was unexpected, given the extensive literature which ascribe the bulge a role in epidermal regeneration 2, 3, 9, 15, 21, 22, 23, 24, 25, 26, 27, 28, 29, 30, 31, 32, 33, but supports the most recent study by Driskell et al., who demonstrated that K19^+ve^ cells are dispensable for IFE re‐epithelialization 10 days postinjury 15. Strikingly, repeated BrdU pulse during the final 3 days of a 7 day healing time‐course produced a similar result: proliferation in numerous IFE and HF keratinocytes, but no proliferation within the bulge (in both anagen and telogen) (Supporting Information Fig. S2).

### Bulge Cells in Peri‐Wound HFs do not Appear to Migrate from the Niche to Contribute to Healing

Although bulge cells in peri‐wound HFs did not proliferate, it was still possible that bulge SCs were migrating out of the bulge compartment in response to injury, as HF melanocyte SCs do in response to wounding 25, 27. To address this, we performed a label retention assay and tracked the destination of bulge‐resident DNA label retaining cells (LRCs) following wounding. This pulse‐chase experiment revealed that, immediately prior to wounding LRCs appeared exclusively confined to the bulge, with no BrdU^+ve^ cells in other skin compartments (i.e., the ORS or IFE; Fig. 1H). Over the first 3 days postinjury LRCs remained within the bulge region of peri‐wound HFs and were never observed to migrate to the ORS or the neo‐epidermis (Fig. 1I).

### Bulge SC Apoptosis can be Selectively Induced Using a K15Cre^PR^;DTA Transgenic Line

**Figure 2:**
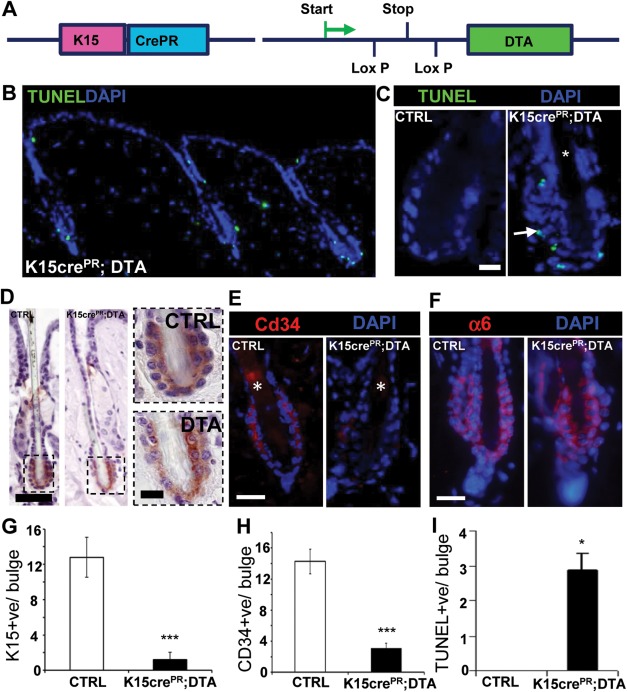
Establishing a K15CrePR;DTA line to conditionally ablate bulge stem cells. **(A)**: A line carrying the RU486 inducible Cre recombinase (CrePR) fused to the Keratin 15 promoter was crossed to a line carrying the DTA, preceded by a floxed stop codon, under the control of a ubiquitous promoter, to create double transgenic K15Cre^PR^;DTA offspring. **(B–D)**: Topical treatment of dorsal skin of K15Cre^PR^;DTA mice with RU486 causes significant apoptosis within the bulge region, while other hair follicle regions remain unaffected. Some apoptosis is observed within the terminally differentiating cells of the epidermis, in both K15Cre^PR^;DTA and wild‐type animals. (D, **G**): Keratin 15 staining reveals a marked reduction in bulge cellularity after RU486 treatment, and a reduction of K15 expression within the stem cell compartment, and **(E, H)** a near absence of Cd34 expression. **(F)**: Reduction in expression of α6 integrin. **(I)**: Number of TUNEL positive cells within the bulge. Graph shows mean ± SEM, * *p* < .05; ***, *p* < .001 scale bar = 20 µm (C, E, F), 40 µm, and 10 µm (D). Abbreviations: DTA, Diphtheria toxin A fragment; DAPI, 4′,6‐diamidino‐2‐phenylindole; TUNEL, Terminal deoxynucleotidyl transferase dUTP nick end labeling.

To functionally probe the importance of the HF bulge for early epidermal repair, we next established an inducible K15‐Cre^PR^;DTA transgenic mouse line which enabled partial temporal depletion of K15^+ve^ bulge SCs (Fig. 2A). As expected, Cre^PR^ activation by topical RU486 administration resulted in major changes to bulge architecture, a marked reduction in K15^+ve^ bulge cells (>70%) and a clear increase in TUNEL^+ve^ cells in the bulge (Fig. 2B–2F). While we were unable to deplete every K15^+ve^ bulge cell (even after daily induction for several weeks; data not shown), cell death was preferentially induced in the Cd34 SC compartment, while the non‐SC K6 compartment remained relatively intact 24 (see Supporting Information Fig. S3). This phenotype broadly mirrors previous attempt at bulge ablation using a genetic approach 34, suggesting ongoing repopulation via another SC pool. While we observe a very low number of TUNEL positive cells within the suprabasal interfollicular epidermis, there is no statistical difference in their number between K15CrePR;DTA and wild type (data not shown).

### Partial Bulge SC Depletion does not Delay Wound Repair

**Figure 3:**
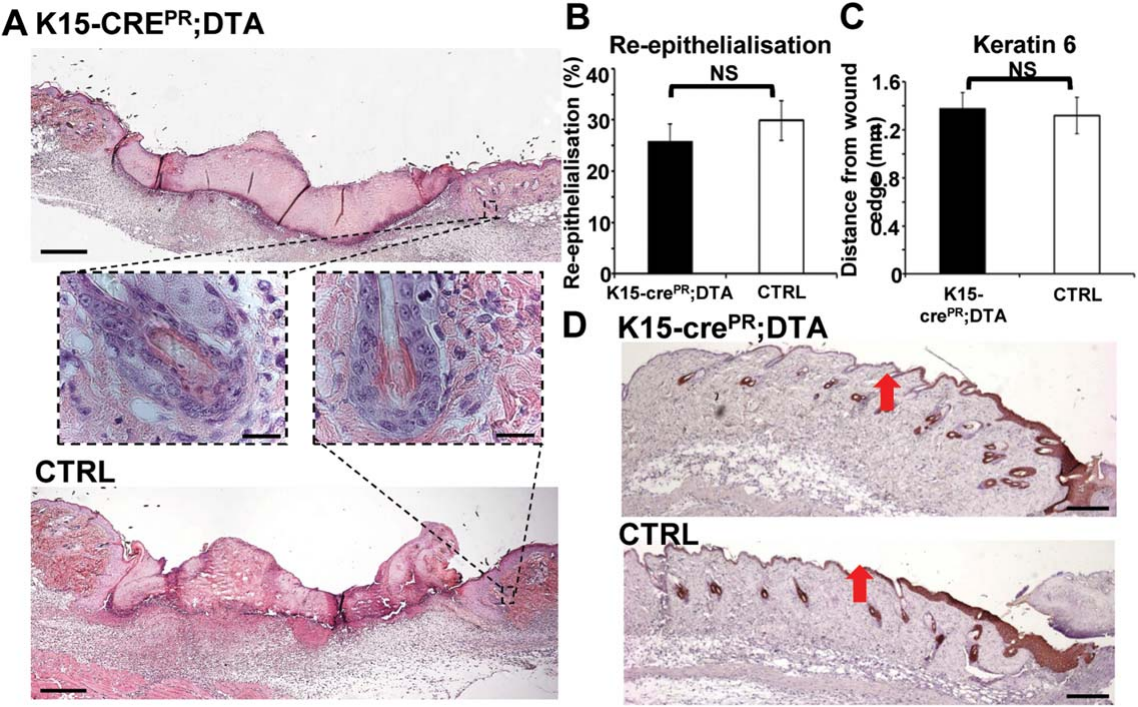
Depletion of bulge stem cells has no effect on early wound healing. K15cre^PR^;DTA and control littermates were wounded after transgene induction. **(A)**: Representative histology indicates equivalent wound size despite clear disruption to the bulge in K15cre^PR^;DTA mice (inset). **(B)**: The rate of re‐epithelialization was unaltered in K15cre^PR^;DTA mice. **(C, D)**: The extent of wound edge keratin 6 expression was unaltered in K15cre^PR^;DTA mice. Mean ± SEM. Scale bar = 300 µm (A), 20 µm (A inset), 100 µm (C).

We next asked whether partial bulge depletion influences wound repair. Intriguingly, ablating the majority of peri‐wound K15^+ve^ HF bulge cells (RU486‐treatment of K15‐Cre^PR^;DTA transgenic mice) prior to wounding (Fig. 3A) had no effect on subsequent wound re‐epithelialization (Fig. 3B). Indeed, we observed no changes in wound healing phenotype using a range of standard wound repair metrics, including granulation tissue area and migration of the regenerated epidermal tongue (Supporting Information Fig. S4A–S4C). Moreover, the extent of wound margin keratin 6 expression, a marker of wound‐responding IFE previously demonstrated to be extended in the absence of HFs, was unaltered in RU486‐treated K15‐Cre^PR^;DTA mice (Fig. 3C, 3D). Taken together, these data indicate that the bulge of unwounded perilesional HFs is not immediately activated by injury or is it required for the initiation of HF‐derived re‐epithelialization.

## Discussion

**Figure 4:**
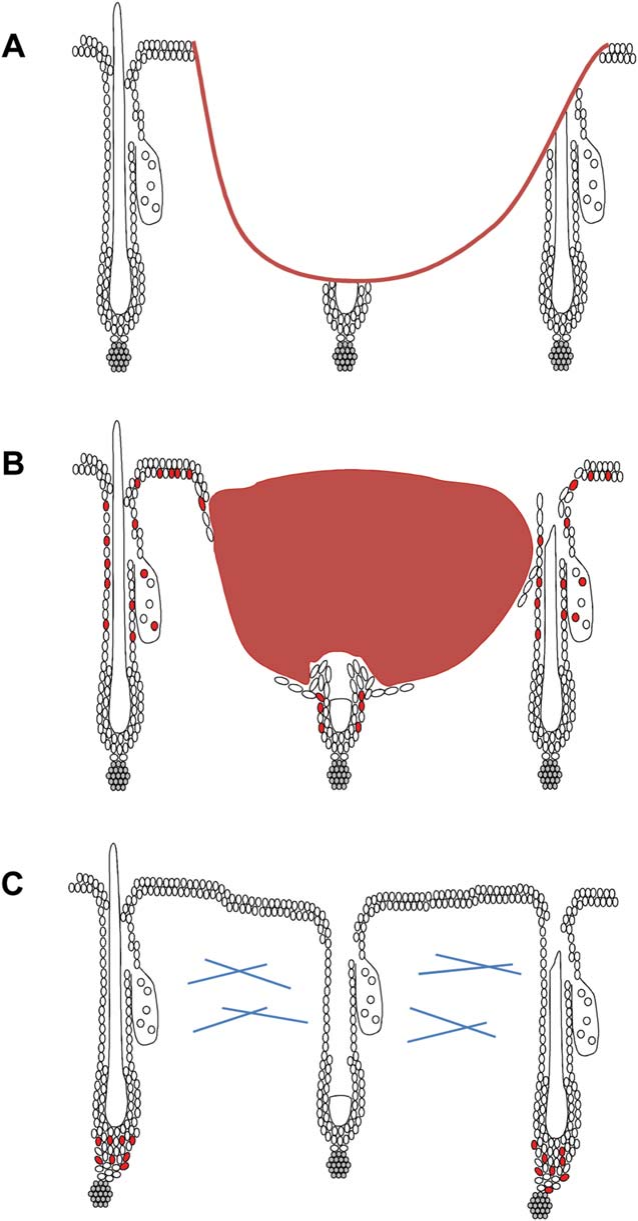
Potential timeline of bulge involvement in wound healing. **(A)**: Cutaneous wound healing can cause direct damage to peri‐wound follicles as well as the interfollicular epidermis (IFE). During the earliest phase of wound healing (0–12 hours postinjury), the hair follicle (HF) response has not yet been initiated, and there are no proliferating cells present within the peri‐wound IFE or HF. **(B)**: From approximately 12–72 hours postinjury, the IFE and HF response is substantial. Peri‐wound HFs display proliferation in the isthmus, infundibulum, and sebaceous glands, but not in the bulge. Bulge cells may be seen to proliferate in HFs that are directly severed as a result of injury. **(C)**: During later phases of healing, peri‐wound HFs have been noted to enter anagen. We propose that hair growth induction during later repair may represent a mechanism whereby bulge cells may proliferate to re‐populate other regions of the HF.

Collectively, our data suggest that wounding does not induce substantial bulge activation in unwounded peri‐wound HFs during the first 3 days of healing. Specifically, bulge cells of uninjured HFs do not proliferate or migrate from the bulge SC niche and partial ablation of K15^+ve^ bulge cells has no measurable impact on the rate of wound healing. While this does not formally exclude that early bulge activation may still occur during other types of epidermal wounding (e.g., burn wounds), when combined with current literature our data strongly suggest that bulge SCs only respond to epidermal wounding during later stages of healing. Indeed, the seminal work of Ito et al. 3, which showed a K15^+ve^ lineage cell contribution to murine neo‐epidermis at 5–8 days postwounding, has been followed by a plethora of studies revealing the influence of HFs on wound repair (Table 1) 2, 3, 7, 9, 10, 11, 15, 35. Our data now suggest differential spatio‐temporal capacity of distinct epithelial HF compartments to respond to wounding. Notably, the bulge appears protected from activation during the early phases of healing, the phase at which HFs provide a functional contribution to repair (Fig. 4) 12, 13.

**Table 1:** Summary of previous studies that have shown bulge contribution to healing

Bulge marker	Early bulge response	Type of study	Brief summary	Reference
K15	Yes	Lineage tracing	Lineage tracing of K15^+ve^ cells to the epidermis at 7–10 days post‐full‐thickness injury. Cells appear from 2 days.	Ito et al. 3
H2B‐GFP (LRC)	Yes	Lineage tracing	Bulge proliferation occurs at 24 hours postinjury (abrasion wound model).	Tumbar et al. 35
H2B‐GFP (LRC)	Yes	In situ analysis	GFP labeled bulge cells become activated 24 hours postinjury (shallow wound model).	Adam et al. 36
Gli1	Unclear	Lineage tracing	No contribution from the lower bulge/hair germ. Lineage tracing to the epidermis from an upper HF region, which might be bulge or isthmus cells.	Brownell et al. 2
K19 (Setd8^−/−^)	No	Lineage tracing	Bulge cells do not enter cell cycle after injury. Blocking bulge cell proliferation does not affect wound repair.	Driskell et al. 15
Lgr5	No (but does respond later)	Lineage tracing	No lineage tracing from the bulge occurs during early repair (3 days) but does occur in later healing (10 days).	Page et al. 9
Lhx2	No (only from 3–5 days)	In situ analysis	Proliferation seen within the bulge at 3–5 days postinjury.	Mardareyev et al. 37

Abbreviations: GFP, green fluorescent protein; HF, hair follicle; LRC, label retaining cell.

We recognize that our observations may be seen to conflict with earlier reports by Tumbar et al. and Mardareyev et al. 35, 36 reporting some bulge proliferation during the first 72 hours of healing. Likewise, Adam et al. 37 reported rapid downregulation of SC transcription factors in the K19^+ve^ bulge SC population prior to injury‐induced migration. However, we suggest that these findings could be the result of analysing directly injured HFs, where bulge proliferation/migration may be specifically induced to regenerate the HF itself 38, rather than the epidermis (Fig. 4B). Alternatively, these studies do not formally exclude the possibility that HFs were in the very early stages of anagen at the time of wounding, when the bulge niche is naturally proliferative. Importantly, in this study, we have selectively measured only uninjured peri‐wound HFs and have used unwounded contralateral skin to control for subtle changes in hair cycle, thus excluding all of these confounding parameters.

In support of our data, Page et al. 9 did not observe any migration of Lgr5^+ve^ labeled cells from the bulge SC niche 2 days after wounding. In fact, Page et al. report that the initial response to injury is by Lrig1 positive cells in the junctional zone, where we also observe high levels of cell proliferation (Fig. 1). Our observation that healing appears to occur independently of the bulge in the first 3 days is further supported by lineage tracing studies where neither Gli1^+ve^ or K19^+ve^ bulge subsets contributed to IFE re‐epithelialization 2, 15. Indeed, epithelial progenitor cells within the isthmus, where we observe the highest level of wound‐induced ORS keratinocyte proliferation, have previously been suggested to be more important for the HF wound response than the bulge 7.

Several papers have reported bulge cell proliferation during later healing 3, 9, 37, 38, most likely the result of a late stage peri‐wound telogen to anagen transition (Fig. 4C) 26. While our study does not address a role for the bulge in later phases of wound repair, we suggest that the bulge's main role during this phase is to act in a secondary manner to replenish epithelial HF populations (such as those from the isthmus region of the ORS) that provide the primary source of HF‐derived epithelial cells to the neo‐epidermis. Thus, bulge quiescence during the early phase of wound healing may be important to preserve the HF's epithelial SC reservoir and to thus conserve the HF's regenerative potential, which would otherwise become depleted immediately following injury 39. Thus, retained bulge cell quiescence during early re‐epithelialization may be evolutionarily advantageous.

The maintenance of epithelial SC quiescence shortly after wounding may also serve as a safeguarding mechanism against tumorigenesis 40. Indeed, the signaling milieu that is induced by both injury and HF cycling is known to predispose mammalian skin to tumor formation 41, 42, with tumour frequency greater during early anagen, that is, when bulge epithelial SCs briefly proliferate 43, 44. Similarly, the formation of squamous cell carcinomas is enhanced following burn injury 45, and injury‐induced basal cell carcinoma formation can be initiated by expressing an oncogenic allele of Smo specifically within K15^+ve^ bulge cells 46.

Indeed, a number of studies have now linked bulge SCs to carcinogenesis namely to the development of basal carcinoma 47, 48, 49. Of note, Petersson et al. 47 observed particularly aggressive tumour formation following aberrant K15‐mediated Lef1 expression 47. If, as suggested, K15^+ve^ cells are particularly sensitive to changes in Lef1 47 or other factors produced by a “pro‐tumorogenic” wound environment, then bulge quiescence during the early, particularly vulnerable proliferative phase of wound healing may also have evolved as a critical tumor‐prevention strategy.

## Conclusion

We conclude that HFs respond rapidly and substantially to skin injury. However, during the earliest phase of wound healing, K15+ve bulge cells are excluded.

## Author Contributions

C.L.G.: concept and design, collection and assembly of data, data analysis and interpretation, manuscript revisions, manuscript writing. D.M.A.: concept and design, collection and assembly of data, data analysis and interpretation, manuscript editing. D.J.H.: provision of study material, concept and design. R.P.: concept and design, manuscript editing. M.J.H.: finance, concept and design, manuscript writing, final editing and approval of manuscript.

## Disclosure of Potential Conflicts of Interest

The authors indicate no potential conflicts of interest.

## Supporting information

Supporting Information

Supporting Information

Supporting Information

Supporting Information
