# Supplementary figures and images for: Hair Follicle Bulge Stem Cells Appear Dispensable for the Acute Phase of Wound Re‐epithelialization
Source: Stem Cells. 2016 Feb 2;34(5):1377–85. doi: 10.1002/stem.2289 (PMC4985639; doi:10.1002/stem.2289)

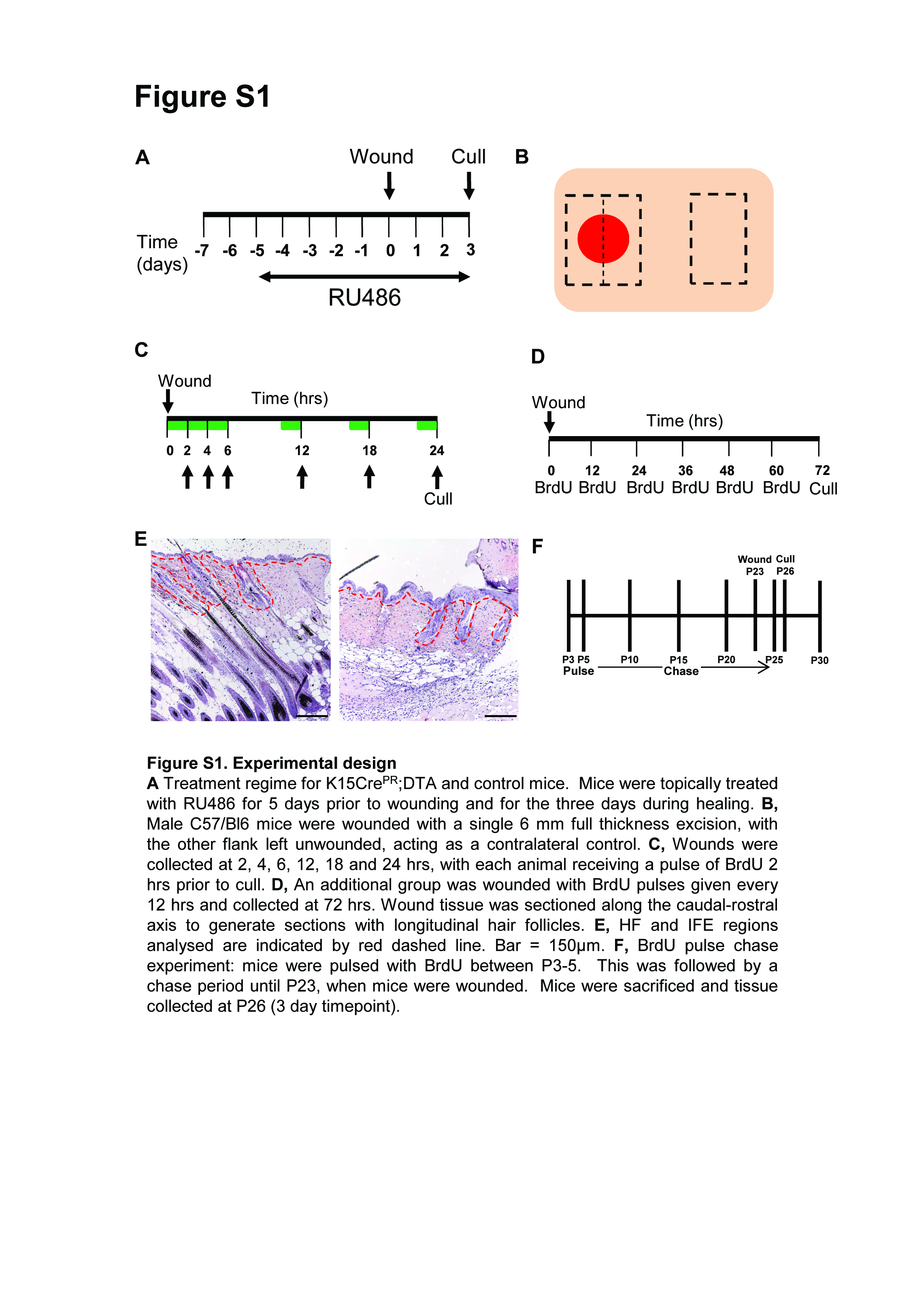

Supplement: Supplementary file 1 — Supporting Information [file STEM-34-1377-s001.tif]

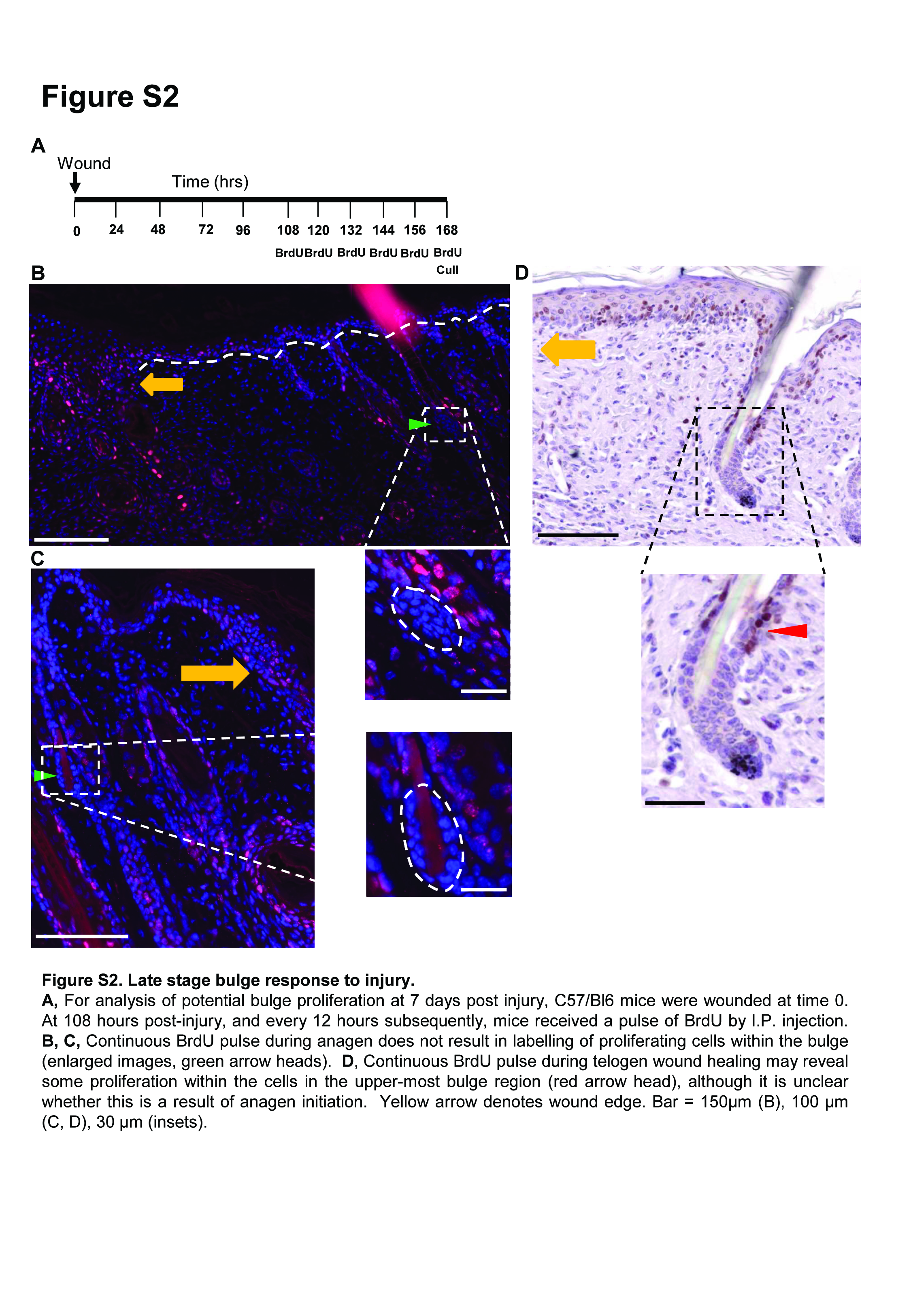

Supplement: Supplementary file 2 — Supporting Information [file STEM-34-1377-s002.tif]

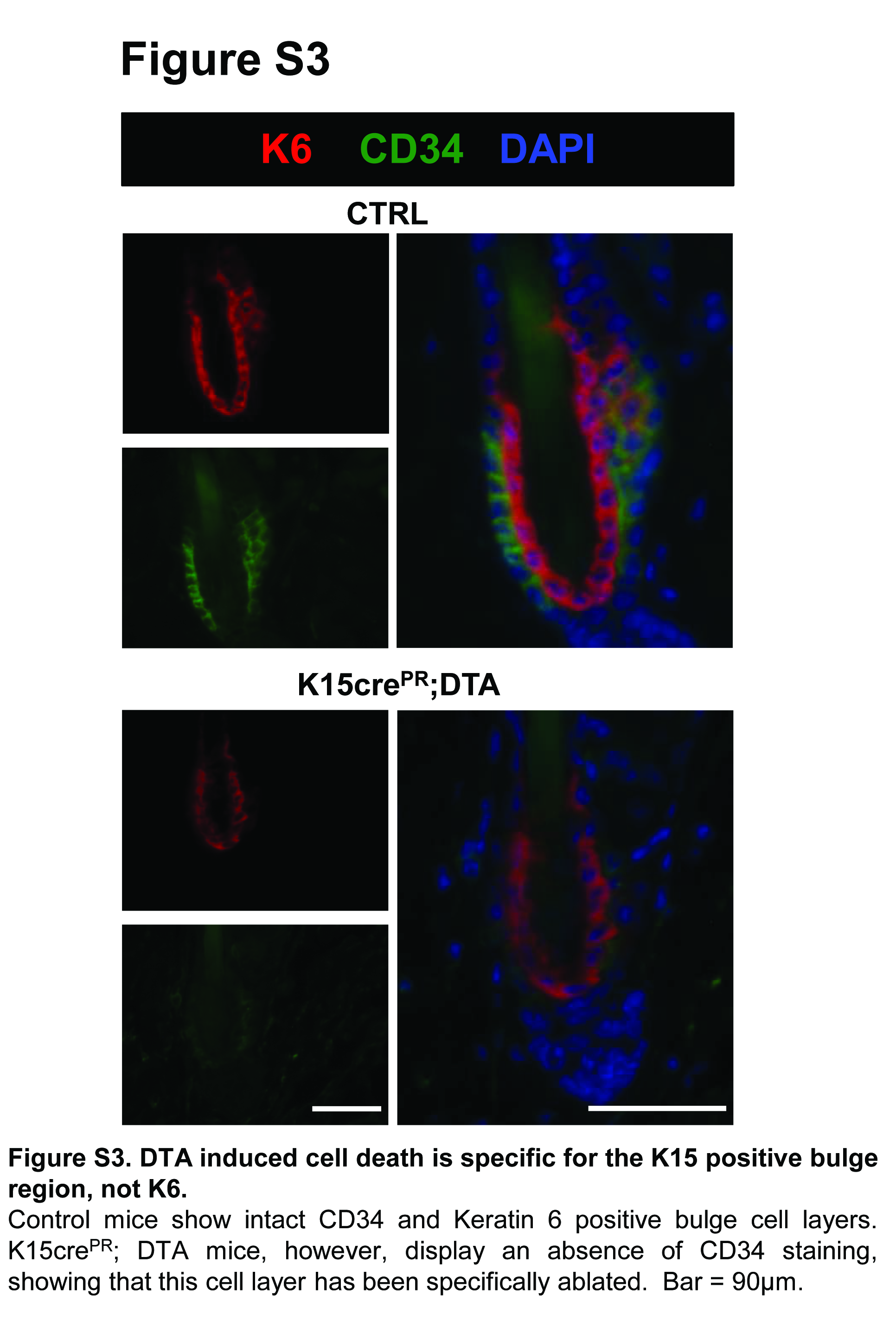

Supplement: Supplementary file 3 — Supporting Information [file STEM-34-1377-s003.tif]

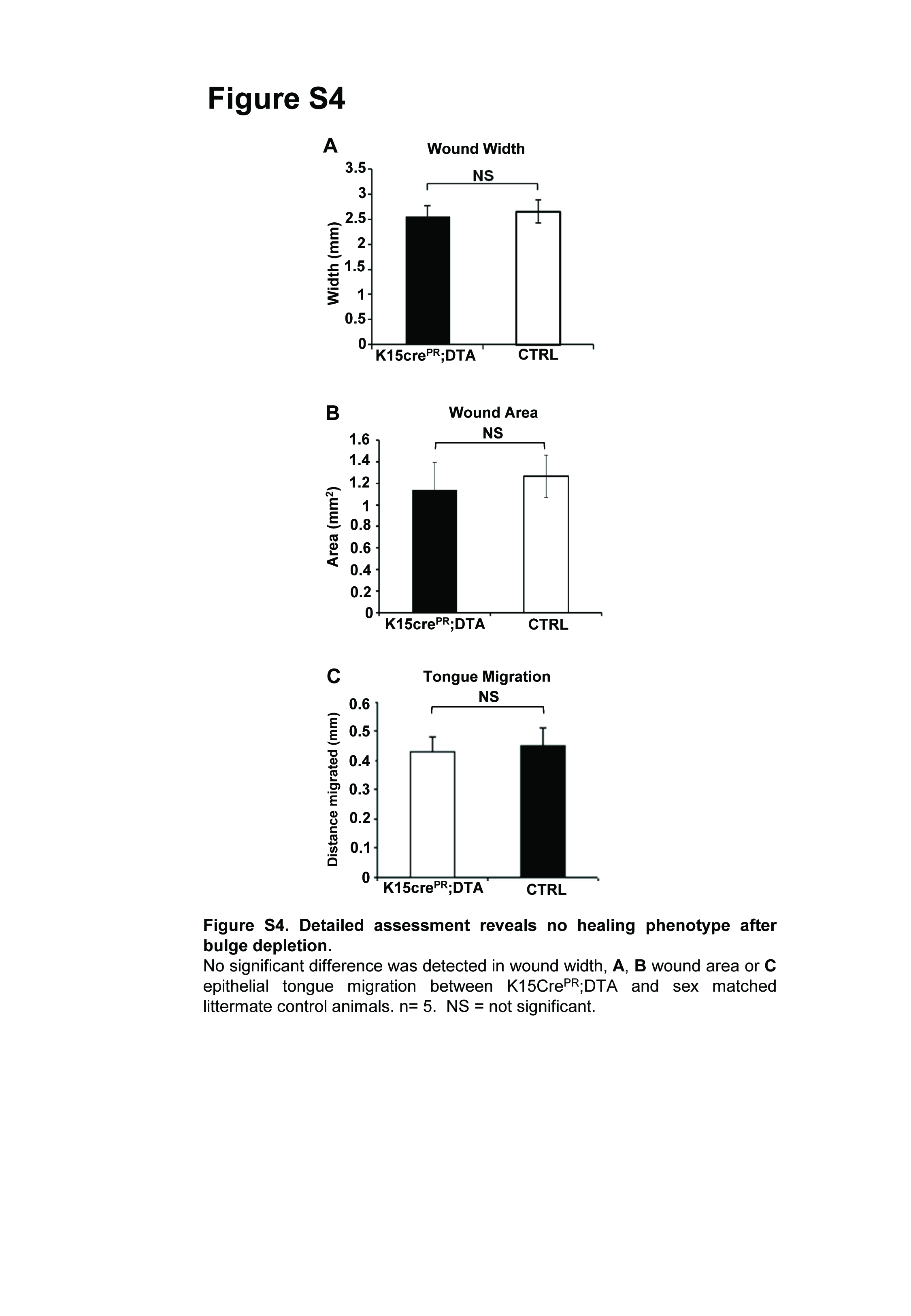

Supplement: Supplementary file 4 — Supporting Information [file STEM-34-1377-s004.tif]
